# Long-Term Outcomes of Abdominal Wall Reconstruction with Expanded Polytetrafluoroethylene Mesh in Pediatric Liver Transplantation

**DOI:** 10.3390/jcm10071462

**Published:** 2021-04-02

**Authors:** Jiyoung Kim, Jeong-Moo Lee, Nam-Joon Yi, Suk Kyun Hong, YoungRok Choi, Kwangpyo Hong, Eui Soo Han, Kwang-Woong Lee, Kyung-Suk Suh

**Affiliations:** Department of Surgery, Seoul National University Hospital, Seoul 08826, Korea; ajykim7@gmail.com (J.K.); gsleejn@gmail.com (N.-J.Y.); nobel1210@naver.com (S.K.H.); choiyoungrok@gmail.com (Y.C.); gigsfire@gmail.com (K.H.); uishann@gmail.com (E.S.H.); kwleegs@gmail.com (K.-W.L.); kssuh2000@gmail.com (K.-S.S.)

**Keywords:** pediatric liver transplantation, abdominal compartment syndrome, Gore-Tex, abdominal wall reconstruction

## Abstract

Background: Large-for-size syndrome caused by organ size mismatch increases the risk of abdominal compartment syndrome. Massive transfusion and portal vein clamping during liver transplantation may cause abdominal compartment syndrome (ACS) related to mesenteric congestion. In general pediatric surgery—such as correcting gastroschisis—abdominal wall reconstruction for the reparation of defects using expanded polytetrafluoroethylene is an established method. The purpose of this study is to describe the ePTFE-Gore-Tex closure method in patients with or at a high risk of ACS among pediatric liver transplant patients and to investigate the long-term prognosis and outcomes. Methods: From March 1988 to March 2018, 253 pediatric liver transplantation were performed in Seoul National University Hospital. We retrospectively reviewed the cases that underwent abdominal wall reconstruction with ePTFE during liver transplantation. Results: A total of 15 cases underwent abdominal closure with ePTFE-GoreTex graft. We usually used a 2 mm × 10 cm × 15 cm sized Gore-Tex graft for extending the abdominal cavity. The median follow up was 59.5 (17–128.7) months and there were no cases of ACS after transplantation. There were no infectious complications related to ePTFE implantation. The patient and graft survival rate during the study period was 93.3% (14/15). Conclusions: Abdominal wall reconstruction using ePTFE is feasible and could be an alternative option for patients with a high risk of ACS.

## 1. Introduction

Liver transplantation (LT) is an important treatment for acute and chronic liver disease in both adult and pediatric patients. Despite advancements in graft allocation, graft matching challenges, especially allocating the right size for recipients, remain. Several surgical techniques for reducing graft size are required for pediatric patients to avoid size mismatch [1]. The graft-to-recipient weight ratio (GRWR) is widely used as a selection criterion, especially for living donor LT, to ensure donor safety and recipient survival. For most centers, the GRWR threshold is between ≥0.8% and ≤4% [2].

In small-for-size syndrome (SFSS), insufficient graft size (GRWR < 0.8) causes hepatic dysfunction with prolonged hyperbilirubinemia, ascites, prolonged coagulopathy, and elevated liver enzymes [3,4]. By contrast, in large-for-size syndrome (LFSS), hepatic dysfunction occurs due to the large liver graft implantation compared to the relatively small recipient body. This phenomenon has been delineated widely in pediatric transplantation. LFSS exhibits poorly perfused liver parenchyma due to the lack of reperfusion flow, vessel kinking, and mechanical compression by the abdominal wall, which leads to the abdominal compartment syndrome (ACS) [5,6]. Studies using pig models have shown that the large-for-size group had a higher level of liver enzymes and inferior graft survival rates than the control group [7,8]. Bowel congestion due to portal vein clamping and massive transfusion during surgery is also attributed to ACS [9]. High intra-abdominal pressure increases intrathoracic pressure associated with pulmonary complications and prolongs intensive care unit (ICU) stay for intubation. Sometimes, it is necessary to keep the abdominal wall open after the surgery. But this delayed closure may cause adverse effects, such as severe infection, in patients with LT [10]. Therefore, closing the abdominal layer with the appropriate tension to avoid ACS in pediatric liver transplant recipients is essential.

Several attempts have been made to reduce morbidity caused by ACS in large-for-size grafts, such as negative pressure wound therapy and bridging mesh closure [11]. Lafossa et al. introduced abdominal wall closure using a combination of tissue expansion and porcine mesh [12]. Additionally, Seaman reported a case series that used expanded polytetrafluoroethylene (ePTFE, GoreTex, W.L. Gore & Associates, Inc., USA) patches in pediatric liver transplantation that showed safe outcomes after operating. They performed second-look operations to remove patches within the first postoperative week [13]. Gore-Tex patches are widely used for other abdominal wall reconstruction, especially in congenital wall defect diseases such as gastroschisis or omphalocele. However, in the area of organ transplantation, it has been mainly reported in multivisceral transplantation and few cases have been reported in liver transplantation alone. Our center has used PTFE graft for abdominal wall reconstruction to prevent ACS in pediatric liver transplant patients.

The aim of this study is to introduce a method for closing the abdominal wall using Gore-Tex to prevent ACS in large-for-size grafts in pediatric LT. It evaluates the long-term safety of the remaining Gore-Tex patch and investigates whether removing the patch is necessary for transplant patients.

## 2. Materials and Methods

From March 1988 to March 2018, 253 patients aged <18 years underwent LT at the Seoul National University Hospital. We retrospectively reviewed 15 cases that underwent abdominal closure with ePTFE-Gore-Tex during LT.

Clinical data—including sex, age, underlying liver disease, Graft-versus-Recipients weight ratio (GRWR), Child–Turcotte–Pugh class, Pediatric Model for End-Stage Liver Disease (PELD) score before transplantation, ascites before transplantation, and previous abdominal surgery history—were evaluated preoperatively. Intraoperative data of estimated blood loss, operative time, and size of the ePTFE graft, were analyzed.

### 2.1. Surgical Technique for Abdominal Wall Reconstruction with ePTFE (Gore-Tex)

**Figure 1:**
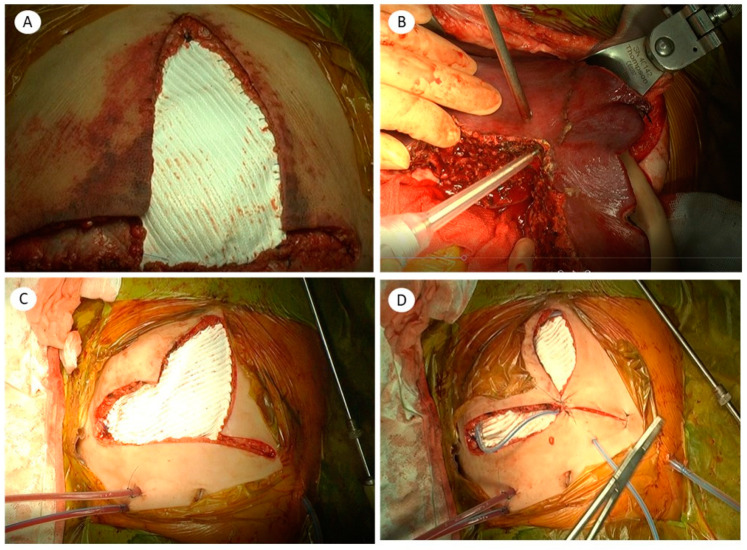
Abdominal wall reconstruction using the expanded polytetrafluoroethylene (ePTFE-Gore-Tex) graft in pediatric liver transplantation: (**A**) reduced expanded polytetrafluoroethylene (ePTFE) graft after a second-look operation after liver transplantation; (**B**) to prevent ACS, liver parenchymal tissue was resected for using reduced graft after reperfusion; (**C**) the fascial layer was closed with the ePTFE graft; (**D**) subcutaneous layer closure and closed suction drain was applied between the skin and fascial layer.

We dissected the subcutaneous layer to make a flap. We pulled the fascial layer as much as possible to reduce fascial defects and secure space for the abdominal cavity. We used a 2 mm × 10 cm × 15 cm sized Gore-Tex graft for extending the abdominal cavity. If possible, the peritoneum was closed and then Gore-Tex was designed and fixed on the fascial layer with a 2-0 prolene suture. After the patch was positioned, the defect was circumferentially closed by continuous suturing between two 2-0 prolene sutures using a non-absorbable material. The edge point of the mesh was reinforced by an interrupted 2-0 vicryl suture to prevent the tearing of the sutured edges. After a closed suction drain was placed between the mesh and subcutaneous layer, the mobilized skin and subcutaneous tissue were approximated en bloc by single suturing with non-absorbable suture material (Figure 1).

### 2.2. Postoperative Evaluation and Follow-up after LT

Recipients were admitted to the intensive care unit routinely for one week after transplantation and administered tacrolimus and steroids as dual immunosuppressants. Patients were evaluated once daily for seven days and twice weekly during the hospital stay through post-transplant liver Doppler sonography. Aspirin was administered to prevent vascular thrombosis. Recipients underwent dynamic computed tomography (CT) 1–2 weeks after the transplant to evaluate graft perfusion, fluid collection, anastomosis, and graft regeneration. If no immediate postoperative complication occurred, then the recipient was discharged 2–3 weeks after transplantation. The steroids were tapered off over six months after transplantation.

### 2.3. Statistical Analyses

The results were expressed as means and standard deviations (SD) or as numbers and percentages. Continuous variables were compared using the Mann–Whitney U-test, and categorical variables were compared using the chi-square test and Fisher’s exact test as appropriate. Data were analyzed using the SPSS 23.0 software (version 23; SPSS Inc., Chicago, IL, USA) and a *p*-value of less than 0.05 was considered statistically significant.

## 3. Results

Over the past 30 years, 253 pediatric LTs were performed at the Seoul National University Hospital. Of these, abdominal reconstruction was performed using an ePTFE Gore-Tex patch in 15 (5.92%) cases (M:F = 8:7). Table 1 shows the perioperative findings of 15 recipients who underwent abdominal wall closure with ePTFE. Recipients underwent transplantation with a split-deceased donor LT (DDLT) (*n* = 8, 53.3%), a whole liver DDLT (*n* = 1, 6.7%), and a living donor liver transplantation (*n* = 6, 40%). In 4 cases of LDLT, a left lateral graft was used. A diagnosis of liver disease comprised of biliary atresia (*n* = 5, 33.3%), glycogen storage disease (*n* = 2, 13.3%), hyperoxaluria (*n* = 2, 13.3%), fulminant hepatic failure (*n* = 2, 13.3%), intrahepatic cholestasis (*n* = 2, 13.3%), urea cycle metabolism disorder (*n* = 1, 6.7%), and choledochal cyst (*n* = 1, 6.7%). Three patients with biliary atresia and one with choledochal cyst had a history of abdominal surgery before transplantation. All patients had ABO-compatible transplantation. Mean GRWR was 2.40 ± 1.1, and mean Pediatric End-stage Liver Disease (PELD) score was 20.5 ± 13.8. In seven cases, retransplantation was performed due to primary graft dysfunction, in which the Gore-Tex patch was used for the second operation. Two of the retransplant cases experienced hepatic failure after the first operation caused by ACS.

Table 2 summarizes the postoperative outcomes of recipients. The median follow-up duration was 59.5 (17–128.7) months, the mean hospital stay was 50.1 ± 47.1 days, and the mean ICU stay was and 17.8 ± 11.5 days. The median estimated blood loss was 300 (100–13,250) cc, and the mean operative time was 365.5 ± 77.6 min. Overall, patient survival rates were 93.3% during follow-up periods.

Only two patients showed a GRWR > 4%, which could be considered large-for-size grafts. The most prolonged ICU stay was for a six-month-old patient who had undergone split DDLT, with a GRWR of 4.19%, and an ICU stay of 43 days (case 2). There was no patient who experienced ACS after transplantation using Gore-Tex for abdominal closure.

**Figure 2:**
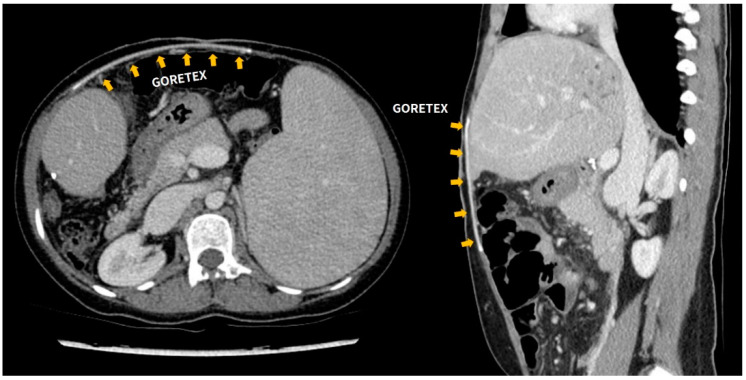
Follow-up abdominal CT scan of patient number 10. Expanded polytetrafluoroethylene (ePTFE-Gore-Tex) graft located between the skin and fascial layer.

Only two (2/15, 13.3%) cases of Gore-Tex-related complication were reported. Case 10 underwent a second LDLT with Gore-Tex closure due to a primary non-functioning graft. After the second transplantation, a cramping pain of unknown origin occurred, which subsided after administering intravenous opioids. This patient continues to have Gore-Tex in her abdominal wall (Figure 2), but no complication was reported in the outpatient clinic follow-up.

**Figure 3:**
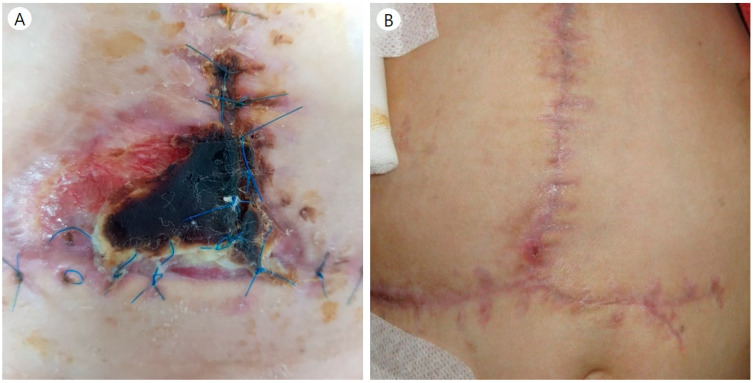
(**A**) Wound complication in patient number 11. (**B**) The plastic surgeon debrided the necrotic skin tissue and reconstructed using a skin flap.

Case 11 underwent a second split DDLT due to the first-graft failure caused by ACS. In the second operation, we used Gore-Tex to ensure a larger intra-abdominal cavity. However, skin necrosis and wound dehiscence around the umbilicus occurred eventually. The plastic surgeon performed a wound revision using a skin flap (Figure 3). In four cases, patients had to undergo repetitive exploration due to complications other than those that were Gore-Tex-related, such as bleeding or vessel occlusion. During the reoperation, we tried to reduce the Gore-Tex graft size as much as possible. Seven patients continue to live with Gore-Tex in their abdominal walls. During long-term follow-up, there were no complications related to Gore-Tex.

## 4. Discussion

A small abdominal cavity and a relatively large transplanted organ in children can lead to ACS during organ transplantation. In 2013, the World Society of the Abdominal Compartment Syndrome (WSACS) consensus guidelines standardized definitions and management of intra-abdominal hypertension (IAH). In children, IAH is defined as IAP ≥ 10 mm Hg [14]. Excessively high IAP that reduces arterial inflow and venous return and thereby endangers abdominal organs’ viability constitutes a condition known as ACS, which is diagnosed in 0.7–4.7% of children treated in the Pediatric intensive care unit (PICU). It is significantly associated with high mortality (40–80%) in critically ill pediatric patients [15]. Impaired arterial and portal blood flow due to elevated IAH can lead to graft loss and patient mortality.

The ideal ratio for liver transplant GRWR is 0.8–2.0% of the recipients’ weight [16]. When this ratio is >4%, there may be problems due to large-for-size transplants, especially in recipients <10 kg [17]. Patients with pediatric LT and multiple organ transplants are at risk for ACS. Most multivisceral transplant candidates have lost abdominal wall secondary to enterocutaneous fistulas or in wound complications due to prior surgery or procedure. It may be impossible to close the abdominal wall and reestablish the peritoneal cavity during transplantation. Therefore, several methods for closing the abdominal wall have been introduced in pediatric multivisceral transplantation procedures.

Usually, when the primary fascia closure is difficult, such as in trauma surgery, many surgeons have tried to keep the wound open in an attempt to delay closure until after the bowel edema improves. In addition, the abdominal wall can be reconstructed by harvesting the abdominal wall of a deceased donor and using it as a free flap [18]. Thus, all methods aim to reduce abdominal wall tension and prevent the occurrence of IAH. However, there is a disadvantage in that the risk of infection is increased for patients who use high doses of immunosuppressants, such as in transplant surgery.

When temporary closure is performed with a mesh, the patient is brought to the operating room a few days after transplantation for the staged closure of the abdominal wall. The use of vacuum-assisted closure devices is discouraged in the early postoperative period because they are associated with complications, such as intestinal fistulas. Notably, if a significant tissue defect occurs, abdominal wall transplantation may be the only available option. However, donor abdominal wall harvesting is not always possible to use as an anastomosis of connecting blood vessels is required and problems related to abdominal wall engraftment may occur. In this situation, the Gore-Tex graft can be used as an alternative material for temporary and permanent abdominal wall closure. Moreover, the ePTFE patch can be removed easily and does not adhere to the bowel, and delayed fascial closure was successful in congenital patients [19].

Usually, Glycogen storage disease (GSD) can wait until a good size fit of graft. Also, there were many metabolic disorders in this report. Usually, they can wait until the body size is developed enough to receive good size-matched grafts. But, in our GSD patient (case 1), the size of the liver gradually increased and the organ failure progressed by pressing the blood vessels and the abdominal organs. Clinically, there were sufficient reasons for rushing liver transplantation. It was a 12-month-old child that was bigger than we thought and that is why we did not use reduce graft in the intraoperative period. Even though we used a pediatric whole liver graft, the GRWR was only 1.6. However, when we tried to close the abdominal wall, the blood vessels could have been pressed due to the shape of the liver graft. There have been reports of good results regarding reduced and hyper-reduced grafts in pediatric transplantation. However, in this case, since he was a large pediatric patient akin to an adult, when the hepatic resection was performed using a relatively large liver, the resection area was wide, and the anterior-to-posterior (AP) diameter of the graft was smaller than the recipient’s abdominal cavity. Even if Rt. or Lt. hemihepatectomy was performed, there was still a possibility of vascular compression. Therefore, we used Gore-Tex for abdominal reconstruction for preventing ACS.

Reviewing patients who had problems with Gore-Tex, case 11 underwent a skin flap operation performed by a plastic surgeon and, due to wound complications, he had fragile skin and subcutaneous tissue because he had undergone abdominal operations several times. Another patient complained of a cramping pain of unknown origin (case 10). It was a peculiar case and it was unclear whether Gore-Tex caused it. We did several tests to evaluate the uncontrolled cramping pain, but we could not find any problems, such as gastrointestinal obstruction. However, considering the possibility of pain caused by nerve irritation due to Gore-Tex, we used a high-dose continuous opioid infusion. After treatment, she was free from pain and was discharged after opioid tapering.

In addition, no complications occurred in other patients. As the median follow-up duration was not enough to guarantee the safety of the method, it is unknown what problems they will face in the long-term follow-up. This is similar to patients with omphalocele or gastroschisis who underwent abdominal reconstruction with mesh.

Above, we presented cases of pediatric patients with a successful reconstruction of the abdominal wall after transplantation with the long-term safety of Gore-Tex. As shown in Table 1, in case 7, the GRWR was not exceptionally large, but when the cavity was marginal, the vessel closed after securing a little space through undermining. Therefore, even if the GRWR is not exceptionally large, Gore-Tex could be applied if the abdominal cavity looks tight in intraoperative evaluation.

In conclusion, ePTFE (Gore-Tex) graft is a good alternative for abdominal wall reconstruction in pediatric LT. The ePTFE (Gore-Tex) graft can be used to close the abdominal wall, either temporarily during transplantation or later when the size is reduced because it does not adhere to the surrounding tissue and can be easily removed. Moreover, if it is placed on the abdominal wall for a long time, no complications related to ePTFE graft are observed.

## Figures and Tables

**Table 1:** Preoperative findings of recipients who underwent abdominal wall reconstruction using the expanded polytetrafluoroethylene (ePTFE).

Case	Sex	Age (mo) at LT	Type of LT	Graft Type	Diagnosis	Blood Type	Retransplantation	PELD	CTP Score	Previous Operation	Ascites before LT
1	F	12	DDLT	Whole liver	Glycogen storage disease	AB+	Y	6.43	A	Y	N
2	M	6	DDLT (SLT)	Lt.lat.	Biliary atresia	AB+	Y	16.67	B	Y	N
3	M	9	LDLT	Lt.lat.	Fulminant hepatic failure	B+	N	57.72	C	N	N
4	F	17	LDLT	Rt.liver	Urea cycle metabolism disorder	B+	Y	25.04	B	Y	N
5	M	8	LDLT	Lt.lat.	Intrahepatic cholestasis	A+	N	15.05	B	N	N
6	F	19	DDLT (SLT)	Lt.lat.	Biliary atresia	A+	N	22.45	B	N	N
7	M	12	DDLT (SLT)	Lt.lat.	Biliary atresia	B+	Y	23.28	C	Y	Y
8	M	10	DDLT (SLT)	Lt.lat.	Glycogen storage disease	B+	N	1.98	A	N	N
9	F	5	DDLT (SLT)	Lt.lat.	Fulminant hepatic failure	B+	N	36.26	C	N	Y
10	F	144	LDLT	Lt.liver	Choledochal cyst	A+	Y	11.9	B	Y	N
11	M	72	DDLT (SLT)	Lt.lat.	Hyperoxaluria	A+	Y	10.37	A	Y	N
12	F	9	LDLT	Lt.lat.	Biliary atresia	AB+	N	18.85	C	Y	Y
13	F	18	DDLT (SLT)	Lt.lat.	Progressive familialintrahepatic cholestasis	B+	N	20.19	B	N	N
14	M	17	DDLT (SLT)	Lt.lat.	Hyperoxaluria	B+	N	6.61	A	N	N
15	M	5	LDLT (SLT)	Lt.lat.	Biliary atresia	O+	Y	36.04	C	Y	Y

LT, liver transplantation; SLT, split liver transplantation; DDLT, deceased donor liver transplantation; LDLT, living donor liver transplantation; PELD, pediatric end-stage liver disease; CTP, Child–Turcotte–Pugh; Lt.lat, Left lateral section; Rt., Right; Lt., Left.

**Table 2:** Post-operative findings of recipients who underwent abdominal wall reconstruction using the expanded polytetrafluoroethylene (ePTFE).

Case	GRWR	ICU Stay (Days)	Operative Time (Minute)	EBL (cc)	Hospital Stay (Days)	Gore-Tex Related Complication	Graft Survival	Patient Survival	OS (Days)
1	1.66	21	455		55	N	Y	Y	3926
2	4.19	43	300	50	61	N	Y	Y	3735
3	3.41	7	410	210	52	N	Y	Y	3364
4	1.62	41	415	2500	121	N	Y	Y	3111
5	2.27	11	415	530	16	N	Y	Y	2738
6	2.72	17	405	280	39	N	Y	Y	2591
7	3.75	10	505	300	24	N	Y	Y	2498
8	1.03	7	420	200	23	N	Y	Y	1816
9	4.44	17	325	620	17	N	N	N	17
10	0.96	30	310	1200	195	Y ^†^	Y	Y	1135
11	1.04	18	240	100	97	Y ^‡^	Y	Y	962
12	2.22	5	323	730	25	N	Y	Y	312
13	2.06	20	390	100	29	N	Y	Y	273
14	2.1	11	360	1000	32	N	Y	Y	150
15	2.6	9	210	300	40	N	Y	Y	124
	2.42 ± 1.08	17.8 ± 11.4	365.5 ± 77.6	300 ^a^ (200–730)	55.1 ± 47.1	2/15 13.3%	14/15 93.3%	14/15 93.3%	1816 ^a^ (273–2738)

LT, Liver transplantation; GRWR, graft-to-recipient weight ratio; ICU intensive care unit; EBL, estimated blood loss; ACS, abdominal compartment syndrome; OS, overall survival; ^†^ Retransplantation case due to primary non-function. Unknown-origin cramping pain occurred after retransplantation. The pain disappeared after high dose opioid usage. ^‡^ Retransplantation case due to ACS. Wound complication occurred around the umbilicus. Wound revision was done using a skin flap by the plastic surgeon; ^a^ Median value (interquartile range).

## Data Availability

The datasets generated and analyzed during the current study are available from the corresponding author on reasonable request.
